# The preoperative G8 geriatric screening tool independently predicts survival in older patients with endometrial cancer: results of a retrospective single-institution cohort study

**DOI:** 10.1007/s00432-022-03934-1

**Published:** 2022-02-25

**Authors:** Katharina Anic, Christin Altehoefer, Slavomir Krajnak, Mona Wanda Schmidt, Roxana Schwab, Valerie Catherine Linz, Marcus Schmidt, Christiane Westphalen, Erik Kristoffer Hartmann, Annette Hasenburg, Marco Johannes Battista

**Affiliations:** 1 Department of Gynecology and Obstetrics, University Medical Center, Johannes Gutenberg-University Mainz, Langenbeckstr. 1, 55131 Mainz, Germany; 2 Department of Geriatric Medicine, University Medical Center, Johannes Gutenberg-University Mainz, Mainz, Germany; 3 Department of Anesthesiology, University Medical Center, Johannes Gutenberg-University Mainz, Mainz, Germany

**Keywords:** Endometrial carcinomas, G8 Score, Frailty, Global health status, Prognosis

## Abstract

**Purpose:**

The aim of this retrospective study was to evaluate the prognostic impact of global health status assessment tools in elderly patients with endometrial cancer (EC) on survival.

**Methods:**

Preoperative frailty status was assessed by the G8 geriatric screening tool (G8 Score), Lee Schonberg prognostic index, Charlson Comorbidity index and American Society of Anesthesiologists Physical Status System in women older than 60 years with EC. Univariable and multivariable Cox-regression analyses, as well as Kaplan–Meier survival analyses were performed to determine the prognostic impact. Statistical analyses were adjusted for cancer entity-specific risk factors such as conventional histopathological tumor characteristics and relevant anamnestic life style parameters.

**Results:**

153 patients with all stages of EC who were operated at the University Medical Center Mainz between 2008 and 2019 were included**.** In multivariable analyses, only the G8 Score retained independent significance as a prognostic factor for disease-specific survival (DSS) (HR:4.58; 95% CI [1.35–15.51]) and overall survival (OS) (HR:2.89; 95% CI [1.31–6.39]. 92 patients (61.3%) were classified as G8-non-frail with a significantly increased DSS and OS rate compared to the 58 G8-frail patients (DSS:93.8% vs. 60.8%; *p* < 0.001 and OS:88.2% vs. 49.7%; *p* < 0.001; respectively).

**Conclusions:**

This is the first study demonstrates the substantial clinical and prognostic impact of the G8 Score on survival in elderly women with EC. Assessing the frailty status to estimate the individual vulnerability of elderly cancer patients could be useful in preoperative decision-making to individualize treatment plans such as the surgical radicality and to improve pre- and postoperative morbidity.

**Supplementary Information:**

The online version contains supplementary material available at 10.1007/s00432-022-03934-1.

## Introduction

Endometrial Carcinomas (EC) are the most frequent gynecological malignancies in developed countries. With a prevalence of 1–2% in all women and an incidence peak between the ages of 60 and 70 years (142,000 new cases in the world per year) (Sung et al. 2021). EC has become”the fifth leading cause of death from cancer in women” (Bourgin et al. 2017). The incidence and the associated mortality are currently rising, potentially due to various factors, including an increasing prevalence of obesity and diabetes as well as the decreasing use of progestin-based postmenopausal hormone treatment (Lu and Broaddus 2020) (Cote et al. 2015). To further specify EC and to make decisions concerning the treatment and prognosis of a patient, conventional risk factors such as histopathological tumor characteristics including tumor stage (TNM and FIGO classification), histological type and grading are used (Felix et al. 2010). Moreover, the postoperative residual tumor burden has been found to be a decisive predictor for surgical quality and consequently of the risk of recurrence (Cree et al. 2020), (Emons et al. 2018).

Increased age is often associated with more aggressive and advanced diseases (Driver and Viswanathan 2017), (Dumas et al. 2016), (Saegusa et al. 2002). The incidence of all cancer entities is statistically overrepresented in the group of the elderly with approximately 54% and with 70% mortality (Siegel et al. 2019). The worse outcome of the population older than 65 years appears to be associated with a lesser likelihood to apply all types of standardized oncological investigations and radical operative treatment strategies (Freedman et al. 2017), (Merchant et al. 1996), (Rauh-Hain et al. 2015). However, the role of biological-calendaric age alone as a perioperative prognostic factor remains controversial (Bourgin et al. 2017), (Creutzberg et al. 2000), (Fleming et al. 2011), (Polanczyk et al. 2001), (Quaglia et al. 2009). Therefore, to increase the chance of cure in older women, an adapted standardized therapy algorithm modified to take the special needs of the older adults is required. Multimorbidity, a dysregulated stress response system and decreased physical performance status with representative symptoms such as fatigue, low activity, weight loss, weakness or low gait and physiologic reserves must be taken into account to modify the current treatment guidelines (Lachance et al. 2006), (Alektiar et al. 2003; Walston 2004). Especially in cancer patients, the offered multimodal therapies, as well as the cancer itself are significant additional stressors that challenge the patient’s physiological reserve (Ethun et al. 2017). The increased vulnerability in frail older adults contributes that this population is less able to tolerate acute stressors, e.g., major surgical interventions with the necessity of multivisceral resections (Birkelbach et al. 2019; Clegg et al. 2013). In general, frailty is associated with increased mortality (Hoogendijk et al. 2021).

In fact, the worse prognosis and postoperative outcome of elderly cancer patients appear to depend on multifactorial geriatric conditions (Ahmed et al. 2018). The basis of heterogeneity due to the aging process contains different domains of frailty and deficiency including comorbid conditions and decreased organ-specific physiological reserves (Balducci and Beghe 2000). The objective of an individual preoperative evaluation of the global health status is to identify frail patients expected to have more treatment-related side effects and toxicities (Clark et al. 2016)*.* Currently, two different approaches exist in geriatric oncology to detect frail patients: the intensive multidisciplinary Comprehensive geriatric assessments (CGA) and the concept of a simple screening tool to identify patients who could benefit from a full subsequent detailed CGA (Hamaker et al. 2012). A CGA is a time-consuming systematic process that objectively assesses somatic, functional and psychosocial domains in elderly patients, which contribute to frailty (Honecker et al. 2011). Thus, more comprise screening tools have been developed, such as the G8 geriatric screening tool (G8 Score) which is one of the most used common global health status screening assessment tools (Kenis et al. 2014), (van Walree et al. 2019). The G8 Score proved to be a practical and time-saving preoperative screening test, especially for elderly cancer patients prior to a major abdominal surgical intervention to identify the frail patients who could benefit from a preoperative multidisciplinary assessment (Honecker et al. 2011), (Soubeyran et al. 2011). Besides the G8 Score, other global health status assessment tools exists: the Lee Schonberg prognostic index (Lee-Index), the American Society of Anesthesiologists Physical Status System (ASA PS) and the original Charlson Comorbidity Index (CC-Index) (Mohile et al. 2018), (Hilditch et al. 2003), (Molto and Dougados 2014).

The objective of this single-institution retrospective cohort study was to determine the prognostic influence of various global health status assessment tools on survival in a consecutive group of women older than 60 years of age with EC, who received radical surgical therapy.

## Materials and methods

### Study population

Women with EC older than 60 years of age irrespective of the histopathological tumor characteristics who underwent primary staging operation in the University Medical Center Mainz between 2008 and 2019 were assessed in this study. Standardized staging operations regardless of the surgical approach (traditional laparotomy or minimally invasive techniques) included a hysterectomy, and bilateral salpingo-oophorectomy, with or without pelvic and para-aortic lymph-node dissection, depending on the currently national guidelines. The surgical treatment was followed by adjuvant radiation or chemotherapy, when appropriate. Long-term follow-up data were collected through telephone calls, written inquiries to the patients or their physicians, and by checking the available patient clinical records up to February 2021. The G8 Score, Lee-Index, ASA PS and CC-Index were retrospectively assessed to determine the individual preoperative global health status.

### Global health status assessment tools

The *G8 geriatric screening tool (G8 Score)* established by Bellera et al. in 2011 is one of the most commonly used rapid geriatric screening test (5 min duration). The test is validated in accordance to predict treatment-related toxicity in elderly cancer patients with systematic chemotherapy. The screening aims to identify frail patients, who could benefit from a full CGA (Soubeyran et al. 2011). As an extension of the Mini Nutritional Assessment questionnaire {item 1–7} (Vellas et al. 1999), the G8 Score also consists of the biological-calendaric age {item 8} (Appendix A1) (Bellera et al. 2012),(Bongue et al. 2017),(Hamaker et al. 2014). The G8 Score ranges from 17 points (not at all impaired—G8-non-frail) to zero points (heavily impaired—G8-frail) with the original cut-off value of ≤ 14 points as an indication for frailty (Hamaker et al. 2012).

The *Lee Schonberg prognostic index (Lee-Index)* is a 4-year life span calculator calibrated in community-dwelling adults older than 50 years. The prognostic index combines in 15 selected questions self-reported comorbidities and functional measures with sex and age (Lee et al. 2006), (Yourman et al. 2012).

The *American Society of Anesthesiologists Physical Status System (ASA PS)* is an international commonly used instrument, developed in 1941 by Meyer Saklad et al. with the title “Grading of Patients for Surgical Procedures” to categorize the preoperative medical health status of adult patients (Fitz-Henry 2011). The classification system ranges from ASA 1—healthy patient to ASA 6—a brain-dead patient whose organs are removed for organ donation (Doyle and Garmon 2018).

The *Charlson Comorbidity index (CC-Index)* was developed and validated to measure 1-year mortality risk and burden of disease especially as a predictor of surgical mortality. Sixteen conditions have been included in the CC-Index with varying degrees of severity (from one to six) depending on their association with mortality risk and disease severity. The sum adds up to the total CC-Index with a maximum of five points (Molto and Dougados 2014).

### Data collection

General patient information was gathered from our hospital database including histopathological tumor characteristics (tumor stage (TNM and FIGO), histological grading and subtype), as well as postoperative residual tumor burden recorded into the official documentation of our interdisciplinary tumor conference. Relevant lifestyle parameters such as age, smoking status, medical history and comorbidities were collected from a physician preoperatively. Furthermore, physical performance status, cognitive functioning and nutritional status were evaluated preoperatively by the oncology nurse specialist together with the patient and the accompanying person through a standardized health status self-assessment questionnaire. Furthermore, perioperative clinical and surgical complications were derived according to the International Statistical Classification of Diseases and Related Health Problems (ICD-10) (Organization, 2004). The G8 geriatric screening tool (G8 Score) consists of eight questions with predefined answer options. Seven items correspond with the MNA (Vellas et al. 1999), (Rubenstein et al. 2001), (Guigoz 2006). The several items assessed in the G8 Score are routinely recorded through a standardized health status self-assessment questionnaire in accordance with the MNA as a standard procedure during the presurgical consultation. Adding the missing item “biological-calendaric age” allows us to calculate the G8 Score retrospectively for each individual patient on this basis with the validated cut-off of ≤ 14 points as an indicator for frailty. The routinely utilized and reproducible Lee-Index was modified afterward by following the calculation without the cancer diagnosis. The CC-Index is calculated with the sum of 16 different conditions according to ICD-10 codes (Organization 2004), all recorded from the physician during preoperative consultation and were deposited in the hospital's internal patient database. All patients undergoing elective surgery were examined at the anesthesia preoperative clinic of the Department of Anesthesiology, where the ASA PS was collected.

In case of ambiguous or missing answers, the total score and estimation of the frailty status was not be performed. The determined frailty definitions and subclassifications of the used global health assessment tools with the numbers of analyzed patients were summarized in Table 4.

### Statistical analyses

The manuscript was written in accordance with the STROBE-cohort checklist of the EQUATOR network reporting guidelines (Von Elm et al. 2007).

Statistical analyses were performed using the SPSS statistical software program, version 23.0 V5 R (SPSS Inc, Chicago, IL, USA). Patients’ characteristics were given in absolute and relative numbers (categorical data), and continuous data were reported as mean and standard deviation or median and their quartiles. First, we compared the histopathological tumor characteristics, as valuated and international recognized prognostic parameters, with unique anamnestic life style parameters and postoperative events of the patients, preoperatively classified as G8-frail and G8-non-frail, using the Chi-square test for categorical or ordinal variables. Anamnestic life style parameters were collected on the basis of the patients' self-assessment in comparison to their peers.

The frailty status of all patients was measured in addition to the G8 Score with the three further global health assessment tools and the results were variously categorized to increase the feasibility in clinical work routine: The Lee-Index and ASA PS divide the study population into four groups, of the one part based on their 4-year mortality probability (< 10%, 10– < 20%; 20– < 30%, > 30%) and the patient’s preanesthesia medical comorbidities, of the other part. The CC-Index describes three groups based on their comorbidity status in relation to the expected 1-year mortality. In addition, we dichotomized the age in a group younger and older than the mean age of study population, to assess the usability in a clinical context.

The Cox-proportional hazard regression model was used to determine the prognostic influence of the selected preoperative global health status assessment tools (G8 Score, Lee-Index, CC-Index and ASA PS). Furthermore, other established risk factors like histopathological tumor characteristics (tumor stage [FIGO, TNM], histological subtype and grade as well as postoperative residual tumor burden) on survival were evaluated to control for these potential confounders likely to influence the prognosis. In addition, unique anamnestic life style parameters (smoker status, physical performance and cognitive functioning) and the influence of biological-calendaric age were included in the model. First, univariate Cox-regression analysis for every single variable was performed. Second, variables with a *p* value < 0.05 entered the multivariable Cox-regression analyses with a variable selection via backward elimination. All associations were given as hazard ratios (HR) with their 95% confidence interval (95% CI) and p values. Kaplan–Meier estimations were used to describe progression free survival (PFS), disease-specific survival (DSS) as well as overall survival (OS). All patients routinely received a computer tomography (CT) either preoperatively to determine the clinical tumor stage or, in the case of non-complete resection a CT scan as baseline imaging postoperatively. Consequentially, PFS was defined as the length of time after the primary operation that a patient lives without a relapse. In case of residual tumor burden, PFS was defined as time after surgery till clinical or radiological progression of disease. DSS was defined as the length of time after the primary operation until the death due to EC. OS was measured from the date of operation to the date of death or last follow-up. The log-rank test was used to compare the survival curves. All associations were given as HRs with their 95% CI and p values. A *p* value of < 0.05 was regarded as statistically significant.

### Results

**Fig. 1:**
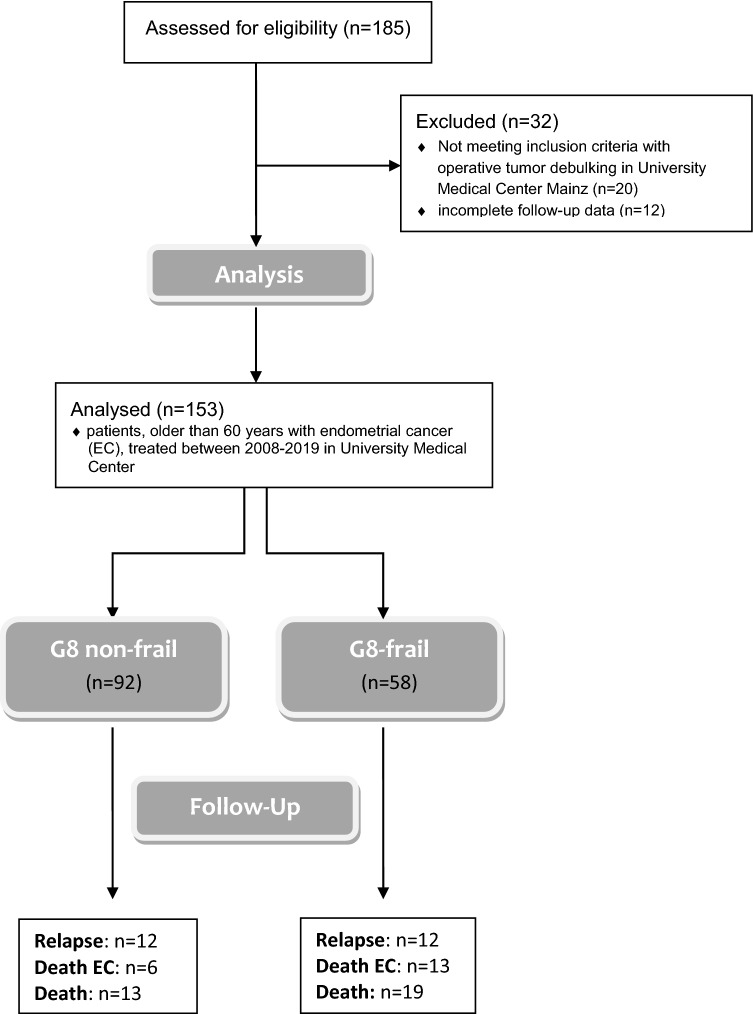
Consort statement

A total of 185 patients were screened. 32 patients were excluded, because they did not undergo primary staging operation in our institution (*n* = 20) or because of incomplete follow-up information (*n* = 12). 153 patients were included in the final retrospective cohort study (Fig. 1). The mean age of the cohort was 71.0 ± 7.4 years. The median follow-up time was 31.0 (8.0–68.5) months. 25 relapses and 20 deaths occurred during the study period (Table 1). All patients were classified with the G8 geriatric screening tool in G8-frail (*n* = 58) and G8-non-frail (*n* = 92), using the previously evaluated cut-off value of ≤ 14 points for being at risk. All conventional histopathological tumor characteristics did not differ between the two cohorts, whereas unique anamnestic life style parameters (e.g., smoker status, physical performance status and cognitive functioning) were statistically more frequent in the G8-frail cohort.

**Table 1:** Characteristics of patients with endometrial cancer (EC)

Parameter (*n*)	*n* (%)	G8-non-frail (*n* = 92; 61.3%)	G8-frail (*n* = 58; 38.7%)	*p* Value^a^
Anamnestic life style parameters				
**Mean age** [years] (± SD) (153)	71.00 (± 7.40)	68.75 (± 6.58)	74.43 (± 7.30)	** < 0.001**
**Smoking status** (145)	13 (9.6)			*0.063*
Smoker and former smokers		5 (6.0)	8 (15.7)	
**Physical performance status** (146)				** < 0.001**
Limited locomotion	23 (16.1)	5 (5.7)	18 (32.1)	
**Cognitive functioning** (146)				**0.002**
Cognitive deficits	9 (6.3)	1 (1.1)	8 (14.3)	
Histopathological tumor characteristics				
**Tumor stage** (TNM, Lewin 2011)				0.122
I	119 (81.0)	77 (85.6)	42 (73.7)	
Ia	68 (46.3)			
Ib	51 (34.7)			
II	7 (4.8)	6 (6.7)	1 (1.8)	
III	11 (7.5)	3 (3.3)	8 (14.1)	
IIIa	2 (1.4)			
IIIb	3 (2.0)			
IIIc1	4 (2.7)			
IIIc2	2 (1.4)			
IV	10 (6.8)	4 (4.5)	6 (10.5)	
IVa	3 (2.0)			
IVb	7 (4.8)			
**Histological grading**				0.439
G1	72 (49.0)	47 (51.6)	25 (44.6)	
G2	46 (31.3)	29 (31.9)	17 (30.4)	
G3	29 (19.7)	15 (16.5)	14 (25.0)	
**Histological subtype**				0.496
Adenocarcinoma	127 (85.2)	79 (86.8)	48 (82.8)	
Others (Endometrioid, Adenosquamous, Mucinous)	22 (14.8)	12 (13.2)	10 (17.2)	
**Postoperative residual tumor burden**				0.562
R0 (no tumor burden)	140 (94.0)	87 (95.6)	53 (91.4)	
R1 (microscopic tumor burden)	3 (2.0)	1 (1.1)	2 (3.4)	
R2 (macroscopic tumor burden)	3 (2.0)	2 (2.2)	1 (1.7)	
Rx	3 (2.0)	1 (1.1)	2 (3.4)	
**Events**				
Relapse	24 (16.1)	12 (13.2)	12 (20.7)	0.224
Death due to EC	19 (12.8)	6 (6.6)	13 (22.4)	**0.005**
Death	32 (21.5)	13 (14.3)	19 (32.8)	**0.007**

Bold values indicate significant results (*p* < 0.05)

Italic values indicate clinically relevant results (*p* < 0.1)

^a^Chi^2^-test

*EC* endometrial cancer, *G8-frail* G8 geriatric screening tool > 14 points, *G8-non-frail* G8 geriatric screening tool ≤ 14 points, *SD* standard deviation, *TNM-FIGO* tumor staging system-international federation of gynecology and obstetrics, *G* histological grading*, R* postoperative residual tumor burden

In the univariable Cox-regression analyses, all examined histopathological tumor characteristics were associated with PFS, DSS and OS except the TNM-tumor stage and the histological subtype for PFS (Table 2). Moreover, preoperative classification as G8-frail was associated with a significantly decreased survival (Table 2 and 3). In contrast, unique anamnestic life style parameters as well as mean age did not predict postoperative survival (Table 2).

**Table 2:** Univariable Cox-regression analyses for survival

	Univariable-PFS			Univariable-DSS			Univariable-OS		
	HR	CI [95%]	*p* value	HR	CI [95%]	*p* value	HR	CI [95%]	*p* value
Histopathological tumor characteristics									
**TNM-tumor stage**	1.48	0.89–2.47	0.134	2.45	1.60–3.74	** < 0.001**	2.21	1.56–3.15	** < 0.001**
**FIGO stage**	1.87	1.34–2.61	** < 0.001**	2.89	2.00–4.19	** < 0.001**	2.25	1.69–2.99	** < 0.001**
**Histological subtype**	0.45	0.18–1.12	*0.087*	0.23	0.09–0.60	**0.002**	0.34	0.16–0.73	**0.006**
**Histological grading**	1.93	1.19–3.12	**0.008**	3.12	1.66–5.90	** < 0.001**	2.26	1.44–3.55	** < 0.001**
**Postoperative residual tumor burden**	2.22	1.06–4.65	**0.034**	3.36	1.93–5.85	** < 0.001**	2.95	1.71–5.08	** < 0.001**
Anamnestic life style parameter									
**Mean age** [71 ys]	1.13	0.53–2.41	0.746	1.36	0.55–3.36	0.500	1.42	0.71–2.84	0.325
**Smoking status**	1.38	0.59–3.21	0.455	0.71	0.13–3.97	0.693	0.80	0.24–2.68	0.719
**Physical performance status**	1.32	0.45–3.85	0.608	0.51	0.23–1.10	*0.087*	2.01	0.81–5.00	0.131
**Cognitive functioning**	0.74	0.10–5.47	0.768	0.49	0.23–1.06	*0.071*	2.29	0.69–7.60	0.177
Global health status assessment tools									
**G8 Score**	2.29	1.04–5.02	**0.040**	4.71	1.76–12.59	**0.002**	3.40	1.67–6.95	**0.001**
**Lee-index**	1.50	1.02–2.21	**0.041**	1.51	0.95–2.41	*0.084*	1.54	1.07–2.22	**0.021**
**CC-index**	1.17	0.68–2.02	0.571	2.23	1.11–4.47	**0.024**	2.21	1.29–3.78	**0.004**
**ASA PS**	1.55	0.78–3.09	0.209	2.05	0.88–4.78	*0.098*	2.26	1.17–4.37	**0.015**

Bold values indicate significant results (*p* < 0.05)

Italic values indicate clinically relevant results (*p* < 0.1)

*PFS* progression-free survival, *DSS* disease-specific survival, *OS* overall survival, *HR* hazard ratio, *CI* confidence interval, *TNM-FIGO* tumor staging system-international federation of gynecology and obstetrics, *G8 Score* G8 geriatric screening tool, *Lee-Index* Lee-Schonberg prognostic index, *CCI* Charlson comorbidity index, ASA PS American society of anesthesiologists physical status system

**Table 3:** Multivariable Cox-regression analyses for survival

	Multivariable-PFS			Multivariable-DSS			Multivariable-OS		
	HR	CI [95%]	*p* value	HR	CI [95%]	*p* value	HR	CI [95%]	*p* value
Histopathological tumor characteristics									
**Tumor stage** (TNM)	n/a			0.84	0.42–1.68	0.612	1.08	0.66–1.78	0.757
**FIGO stage**	1.72	1.19–2.50	**0.004**	2.52	1.68–3.78	** < 0.001**	1.98	1.45–2.71	** < 0.001**
**Histological subtype**	n/a			2.93	0.62–13.89	0.177	2.14	0.63–7.26	0.221
**Histological grading**	1.51	0.84–2.72	0.168	1.74	0.84–3.59	0.135	1.47	0.85–2.56	0.173
**Postoperative residual tumor burden**	1.38	0.53–3.62	0.510	1.21	0.51–2.89	0.671	0.94	0.35–2.51	0.898
Anamnestic life style parameter									
**Mean age**	n/a			n/a			n/a		
**Smoking status**	n/a			n/a			n/a		
**Physical performance**	n/a			n/a			n/a		
**Cognitive functioning**	n/a			n/a			n/a		
Global health status assessment tools									
**G8 Score**	2.01	0.87–4.65	0.102	4.58	1.35–15.51	**0.015**	2.89	1.31–6.39	**0.009**
**Lee-index**	1.12	0.68–1.86	0.654	n/a			1.12	0.69–1.81	0.644
**CCI**	n/a			1.23	0.50–3.02	0.647	1.50	0.77–2.95	0.234
**ASA PS**	n/a			n/a			1.35	0.51–3.54	0.545

Bold values indicate significant results (*p* < 0.05)

Italic values indicate clinically relevant results (*p* < 0.1)

*PFS* progression-free survival, *DSS* disease-specific survival, *OS* overall survival, *HR* hazard ratio, *CI* confidence interval, *TNM-FIGO* tumor staging system-international federation of gynecology and obstetrics, *G8 Score* G8 geriatric screening tool, *Lee-Index* Lee-Schonberg prognostic index, *CCI* Charlson comorbidity index, ASA PS American society of anesthesiologists physical status system, n/a not applicable (excluded because not significant values in univariate Cox-regression analysis)

In the multivariable analyses, only FIGO stage and the preoperative frailty validation with G8 Score retained their independent significance for DSS and OS in patients older than 60 years with EC (G8 – DSS: HR: 4.58; 95% CI [1.35–15.51], G8 – OS: HR: 2.89; 95% CI [1.31–6.39]) (Table 3). All other conventional histopathological tumor characteristics (TNM-tumor stage, histological subtype and grading as well as postoperative residual tumor burden) and further global health status assessment tools (Lee-Index, CC-Index and ASA PS) were not independently associated with the postoperative prognosis (all p values > 0.05).

**Fig. 2:**
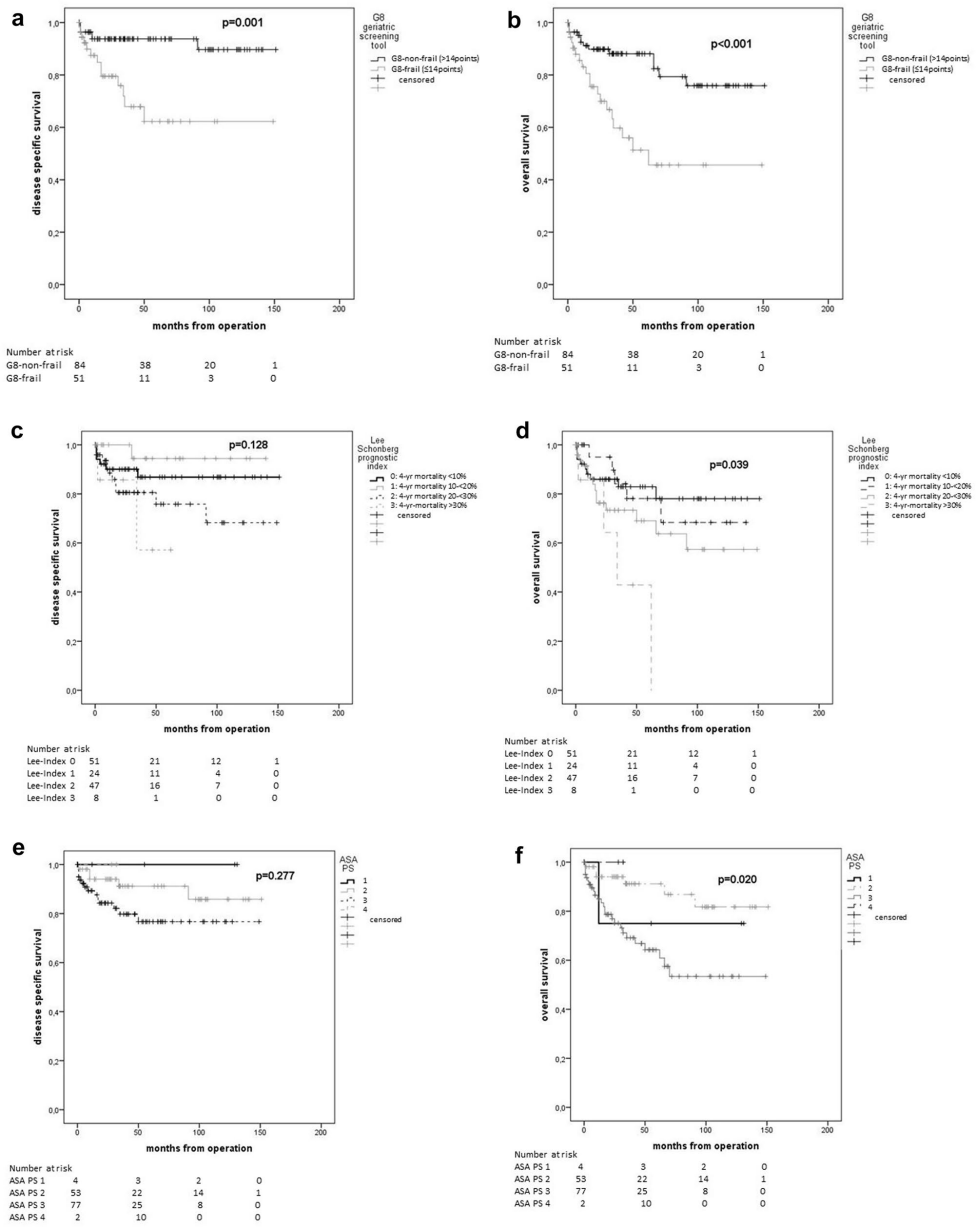

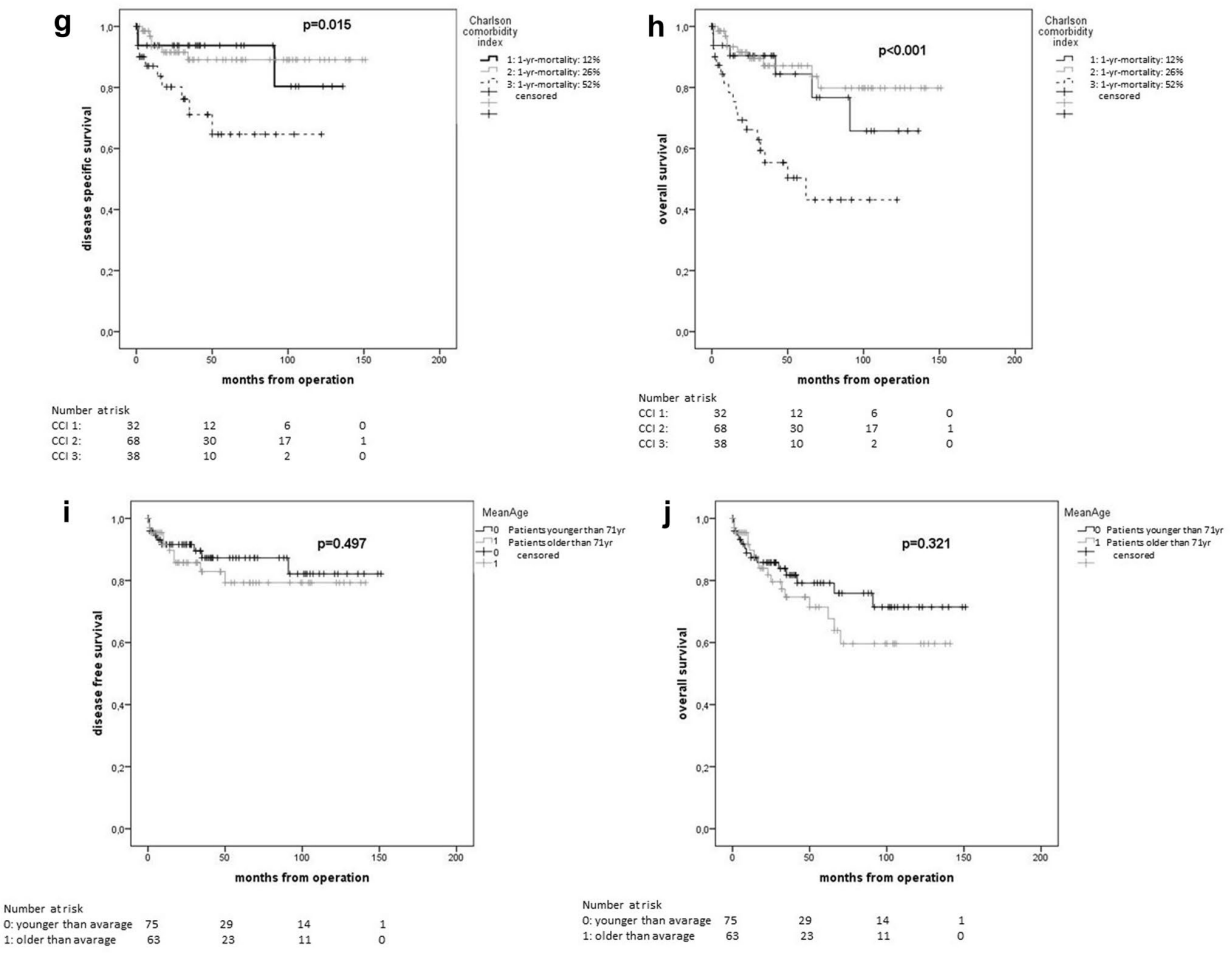
Kaplan–Meier plots of global health status assessment tools and age. G8 geriatric screening tool (**a**), (**b**), Lee Schonberg prognostic index (**c**), (**d**), American Society of Anesthesiologists Physical Status System (**e**), (**f**), Charlson Comorbidity Index (**g**), (**h**), and mean age (**i**), (**j**) for disease-specific survival (**a**, **c**, **e**, **g**, **i**) and overall survival (**b**, **d**, **f, h**, **j**)

In Kaplan–Meier estimations, all global health assessment tools showed statistically significant differences in terms of 5-year OS (Table 4). Mean age showed no significant difference for 5-year OS (younger than average 79.2% vs. older than average 71.4%; *p* = 0.321) (Fig. 2).

**Table 4:** Estimated 5-year survival rates by Kaplan–Meier method in relation to preoperative frailty status

Global health status assessment tools – frailty status classification	*n* (%)	PFS after 5 years [%], *p* value	DSS after 5 years [%], *p* value	OS after 5 years [%], *p* value
**G8 geriatric screening tool (G8 Score)**	150	*0.071*	**0.001**	** < 0.001**
G8-non-frail	92 (61.3)	82.1	93.8	88.2
G8-frail	58 (38.7)	65.4	60.8	49.7
**Lee Schonberg prognostic index (Lee-index)** (4-year mortality [%])	146	**0.039**	0.128	**0.039**
0 (< 10)	57 (39.0	83.1	86.8	82.9
1 (10–< 20)	28 (19.2)	85.3	94.4	78.1
2 (20–< 30)	50 (34.2)	55.9	75.8	69.0
3 (> 30)	11 (7.5)	66.7	57.1	42.0
**American society of anesthesiologists physical status (ASA PS)**	152	0.462	0.277	**0.020**
1	4 (2.6)	75.0	100.0	75.0
2	59 (38.8)	81.4	91.2	91.2
3	87 (57.2)	71.5	76.7	64.3
4	2 (1.3)	0.0	0.0	0.0
**Charlson comorbidity index (CC-Index)** (1-year mortality [%])	154	0.173	**0.015**	** < 0.001**
0 (12%)	0 (0.0)	0.0	0.0	0.0
1 (26%)	37 (24.0)	73.6	93.8	84.4
2 (52%)	73 (47.4)	79.0	89.3	87.3
3 (85%)	44. (28.6)	61.8	64.6	50.4

Bold values indicate significant results (*p* < 0.05)

Italic values indicate clinically relevant results (*p* < 0.1)

*PFS* progression-free survival, *OS* overall survival, *DSS disease-specific survival*

In univariate Cox-regression analyses, four out of eight items of the G8 Score (functional status {item 4}, cognitive status {item 5}, polypharmacy {item 6} and increased age over 80 years {item 8}) predicted OS (HR: 0.47, 95% CI [0.26–0.86]; HR: 0.49, 95% CI [0.27–0.90]; HR: 0.33, 95% CI [0.16–0.71] and HR: 0.43, 95%CI [0.25–0.75]; respectively) (Supplementary Table).

### Discussion

To the best of our knowledge, we report an independent prognostic impact of the G8 geriatric screening tool on DSS and OS for women older than 60 years with all tumor stages of EC for the first time. The preoperatively used G8 Score demonstrated this impact independent of established histopathological tumor characteristics in multivariable analyses. In contrast, other global health assessment tools, e.g., Lee-Index, CC-Index and ASA PS, were not able to stratify the cohort into subgroups with a statistically significant and independent survival difference. Furthermore, various single clinically meaningful anamnestic life style parameters (e.g., smoking status, physical performance status or cognitive functioning) were also not reliable prognostic factors. One reason might be that frailty is nowadays regarded as an age-related complex and multifactorial syndrome of marked vulnerability, which is inadequately reflected by single anamnestic factors (Fried 2001),(Bandeen-Roche et al. 2006). Clegg et al. defined frailty as “the most problematic expression of population aging. It is a state of vulnerability to poor resolution of homoeostasis after a stressor event and is a consequence of cumulative decline in many physiological systems during a lifetime” (Clegg et al. 2013).

Moreover, older cancer patients have been shown to be less able to tolerate and adapt to stressors like acute illnesses or surgical interventions (Linn et al. 1982),(Youl et al. 2021). One could be explained by the excellent prognostic impact of preoperative evaluation with G8 Score because of the combination of items with the main focus on nutrition status, physical performance as well as cognitive function and social status. The G8 Score can specially assess the contribution to daily functioning and patients’ resilience to the major stressor, surgery (van Gestel et al. 2013). Moreover, the relationship between increasing age and frequently associated comorbidities results in loss of physical capacity and > 60% decline in postoperative self-care in older patients (Hamaker et al. 2015).

Our findings are in line with other reports. Driver et al. reported a strong prognostic value for the presence of selected frailty markers including weight loss and BMI < 20 kg/m^2^, as well as ECOG > 2 and laboratory pathologies for 3-year DSS (HR: 2.21; 95% CI: 1.02–4.80) and for a shortened OS (HR: 2.34; 95% CI: 1.08–5.03) in a prospective cohort study with 88 consecutive women with EC in 2017 (Driver and Viswanathan 2017). Decoster et al. postulated in their systematic literature review that geriatric screening tools are recommended to identify those patients in need of further evaluation for a multidisciplinary approach in a busy clinical practice (Decoster et al. 2015). Moreover, in another retrospective cohort study, our study group could demonstrated that the G8 score independently predicted PFS in older ovarian cancer patients regardless of maximal surgical effort (Anic et al. 2021).

To individualize therapy for the very heterogeneous older population, a global health validation with validated geriatric assessment tools to identify frailty status before extended oncological surgery should be applied as opposed to age alone (Nadaraja et al. 2018),(Fried 2001). With reference to the existing evidence of age-related conditions and deficits, and not biological-calendaric age itself, preoperative global health assessment tools should be used to consider risks and benefits of adjuvant cancer treatment. In contrast, a number of studies have shown that increasing age could be a risk factor for poorer tolerance especially regarding adjuvant chemotherapy just as radiation, but data for the predictive power before operations are missing (Nadaraja et al. 2018).

Contrastingly, Nadaraja et al. published a prospective randomized controlled study focused on the systematically oncologic treatment decision based on the G8 Score followed by CGA in women older than 70 years of age with various cancer entities. The results were not be able to show an influence on completion rate of oncologic treatment or survival compared to a therapy decision based on standard assessment (Nadaraja et al. 2020). Reasonably, their findings refer to a small and heterogenous cohort of only 21 G8-frail patients with different cancer entities. Moreover, the presented results concern a cohort with predominantly elderly patients in good health condition with a majority of only grade 1–2 of chemotherapy-related toxicities in their population. Nevertheless, the patients with presurgical geriatric screening had a borderline significantly lower incidence of grade 3–4 toxicity (88% vs. 20%; *p* = 0.055).

Limitations could arise apart from the retrospective and single-institution character of the study from the fact that a small amount of individual data were lost due to the calculation of the frailty assessment tools based on various standardized multiple-choice questionnaires. In case of ambiguous or missing answers, the total score and estimation of the frailty status were not be performed. However, with the completeness of data from 94.8 to 100% (see Tables 1 and 4), the risk of bias due to missing data is minimal. This may be also relevant especially in terms of incomplete follow-up information which was successfully reduced to a minimum of 12 patients by reaching out to patients and physicians through different ways of communication and an extensive review of clinical records. One advantage of our study is the comparability with regards to baseline characteristics of the G8-frail and G8-non-frail cohort. Moreover, the conventional histopathological tumor characteristics, the surgical procedures, the postoperative residual tumor burden as well as surgical parameters were comparable in the G8-frail and G8-non-frail subgroup. These facts underline the *independent* prognostic impact on DSS and OS of the G8 Score in our cohort. In contrast, the biological-calendaric age alone did not predict DSS nor OS after 5 years, although it was established as an independent risk factor in numerous prior publications. This might be explained by the fact that patients younger than 60 years have been excluded from our study, to enlarge the cohort with a higher number of frail patients.

In conclusion, the G8 Score can be used to predict DSS and OS prior to surgery in elderly women with EC. Preoperative assessment and treatment decisions should not solely rely on a simplistic view of biological-calendaric age or anamnestic life style parameters, but should incorporate more sophisticated tools such as the G8 Score. Making individualized treatment decisions for elderly patients based on preoperative global health status assessment tools through standardized screenings would be desirable. Further research should also focus on preoperative interventions to increase the global health status of G8-frail patients and their potential to improve postoperative outcomes.

## Supplementary Information

Below is the link to the electronic supplementary material.

Supplementary file1 (DOCX 17 kb)

## Data Availability

All data generated or analyzed during this study are included in this article and its supplementary material files. Further enquiries can be directed to the corresponding author.

## Abbreviations

ASA PS: American society of anesthesiologists physical status system
CC-Index: Charlson comorbidity index
CGA: Comprehensive geriatric assessments
CT: Computer tomography
95% CI: Confidence interval
DSS: Disease-specific survival
EC: Endometrial cancer
FIGO: International federation of gynecology and obstetrics
G8 Score: G8 geriatric screening tool
HR: Hazard ratio
Lee-Index: Lee Schonberg prognostic index
OS: Overall survival
PFS: Progression-free survival
TNM: Tumor staging system
