# Supplementary material for: The preoperative G8 geriatric screening tool independently predicts survival in older patients with endometrial cancer: results of a retrospective single-institution cohort study
Source: J Cancer Res Clin Oncol. 2022 Feb 25;149(2):851–63. doi: 10.1007/s00432-022-03934-1 (PMC9931812; doi:10.1007/s00432-022-03934-1)
Supplement: Supplementary file 1 — Supplementary file1 (DOCX 17 kb) [file 432_2022_3934_MOESM1_ESM.docx]

**Supplementary: G8 geriatric screening tool**

|  | **Category** | **Items** | **Possible responses (score)** | **HR** | **95%-CI** | **p value** |
| --- | --- | --- | --- | --- | --- | --- |
| **{1}** | Nutritional  status | Has food intake declined over the past three months due to loss of appetite, digestive problems, chewing, or swallowing difficulties? | ⬜**0**: Severe decrease in food intake  ⬜**1**: Moderate decrease in food intake  ⬜**2**: No decrease in food intake | 0.30 | 0.04-2.22 | 0.238 |
| **{2}** |  | Body-Mass-Index (BMI) [kg/m²]? | ⬜**0**: BMI <19  ⬜**1**: BMI between 19-21  ⬜**2**: BMI between 21-23  ⬜**3**: BMI ≥ 23 | 0.59 | 0.33-1.05 | *0.073* |
| **{3}** |  | Weight loss during the last three months? | ⬜**0**: Weight loss >3 kg  ⬜**1**: Unknown  ⬜**2**: Weight loss between 1 and 3 kg  ⬜**3**: No weight loss | 0.58 | 0.28-1.20 | 0.142 |
| **{4}** | Functional  status | Mobility? | ⬜**0**: Bed or chair bound  ⬜**1**: Able to get out of bed/  chair but does not go out  ⬜**2**: Goes out | 0.47 | 0.26-0.86 | **0.013** |
| **{5}** | Cognitive  status | Neuropsychological problems? | ⬜**0**: Severe dementia/depression  ⬜**1**: Mild dementia/depression  ⬜**2**: No psychological problems | 0.49 | 0.27-0.90 | **0.020** |
| **{6}** | Comorbidities | Takes more than three prescripted  drugs per day? | ⬜**0**: Yes  ⬜**1**: No | 0.33 | 0.16-0.71 | **0.004** |
| **{7}** |  | In comparison with other people of the same age, how does the patient  consider his/her health status | ⬜**0**: Not as good  ⬜**0,5**: Does not know  ⬜**1**: As good  ⬜**2**: Better | 0.46 | 0.21-1.01 | *0.053* |
| **{8}** |  | Age? | ⬜**0:** >85 years  ⬜**1:** 80 - ≤85 years  ⬜**2:** <80 years | 0.43 | 0.25-0.75 | **0.003** |

*HR:* **H**azard **R**atio, *95%-CI:* **C**onfidence **I**nterval

**bold written words**: analysed main categories, **bold written numbers**: significant results (p<0.05), *italic written numbers*: clinically relevant results (p<0.1)
